# Plasma nicotinamide adenine dinucleotide pathway depletion is linked to diastolic dysfunction in patients with advanced chronic liver disease

**DOI:** 10.1093/cvr/cvaf181

**Published:** 2025-11-11

**Authors:** Madeleine Gill, John F O’Sullivan, Giovanni Guglielmi, Eugene Slaughter, Ren Ping Liu, Imre Hunyor, Stuart Moss, Michele McGrady, Ian Wilcox, Geoffrey William McCaughan, Avik Majumdar

**Affiliations:** Faculty of Medicine and Health, University of Sydney, Missenden Road, Sydney, NSW 2050, Australia; AW Morrow Department of Gastroenterology and Hepatology, Australian National Liver Transplant Unit, Royal Prince Alfred Hospital, Sydney, NSW, Australia; Liver Injury and Cancer Program, The Centenary Institute, Sydney, NSW, Australia; Faculty of Medicine and Health, University of Sydney, Missenden Road, Sydney, NSW 2050, Australia; Department of Cardiology, Royal Prince Alfred Hospital, Missenden Road, Sydney, NSW 2050, Australia; Faculty of Medicine and Health, University of Sydney, Missenden Road, Sydney, NSW 2050, Australia; Faculty of Medicine and Health, University of Sydney, Missenden Road, Sydney, NSW 2050, Australia; Faculty of Medicine and Health, University of Sydney, Missenden Road, Sydney, NSW 2050, Australia; Faculty of Medicine and Health, University of Sydney, Missenden Road, Sydney, NSW 2050, Australia; Department of Cardiology, Royal Prince Alfred Hospital, Missenden Road, Sydney, NSW 2050, Australia; Department of Cardiology, Royal Prince Alfred Hospital, Missenden Road, Sydney, NSW 2050, Australia; Faculty of Medicine and Health, University of Sydney, Missenden Road, Sydney, NSW 2050, Australia; Department of Cardiology, Royal Prince Alfred Hospital, Missenden Road, Sydney, NSW 2050, Australia; Faculty of Medicine and Health, University of Sydney, Missenden Road, Sydney, NSW 2050, Australia; Department of Cardiology, Royal Prince Alfred Hospital, Missenden Road, Sydney, NSW 2050, Australia; Faculty of Medicine and Health, University of Sydney, Missenden Road, Sydney, NSW 2050, Australia; AW Morrow Department of Gastroenterology and Hepatology, Australian National Liver Transplant Unit, Royal Prince Alfred Hospital, Sydney, NSW, Australia; Liver Injury and Cancer Program, The Centenary Institute, Sydney, NSW, Australia; Faculty of Medicine and Health, University of Sydney, Missenden Road, Sydney, NSW 2050, Australia; Victorian Liver Transplant Unit, Austin Health, Melbourne, Victoria, Australia; Faculty of Medicine, Dentistry and Health Sciences, The University of Melbourne, Melbourne, Victoria, Australia

**Time of primary review: 25 days**

Cirrhotic cardiomyopathy (CCM) is a common yet often subclinical complication of advanced chronic liver disease (ACLD), characterized by diastolic (DD) and systolic dysfunction (SD), chronotropic incompetence and a blunted contractile reserve (*Figure 1A*).^1^ No targeted therapies exist, and its underlying pathophysiology remains poorly understood, particularly in relation to metabolic alterations. As CCM is phenotypically similar to heart failure with preserved ejection fraction (HFpEF), recent insights into disrupted cellular energetics^2^ and nicotinamide adenine dinucleotide (NAD^+^) pathway depletion^3^ in HFpEF prompted this investigation into metabolic dysfunction in CCM.

**Figure 1:**
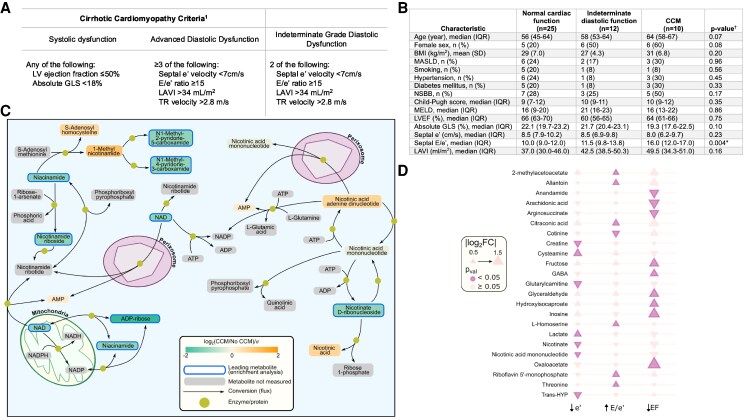
(*A*) Definition of CCM based on the Cirrhotic Cardiomyopathy Consortium criteria.^1^ (*B*) Table of baseline characteristics, comorbidities and cardiac indices in all patients, grouped by normal cardiac function, indeterminate diastolic dysfunction and confirmed CCM. ^†^*P*-value calculated comparing patients with CCM vs. without CCM; *denotes significant *P*-value (*C*) Pathway analysis using Fast Gene Set Enrichment Analysis (FGSEA). The figure represents NAD biosynthesis and salvage pathways and its role in ATP synthesis and as a mitochondrial coenzyme. The green shaded metabolites were downregulated in patients with CCM compared with patients without CCM (NES −1.65; *P* = 0.02). The blue bordered leading metabolites were considered to be significantly drivers of the overall pathway, whereas the metabolites without borders were not implicated. This demonstrates disruption at multiple points in the NAD pathway and suggests alteration of cellular energetics as a potential mechanism for myocardial dysfunction in CCM. (*D*) Bubble plot demonstrating the differences (by log2 FC) in the differential expression of individual metabolites in association with cardiac indices. Note that e′ decreases with diastolic dysfunction while E/e′ increases. ADP, adenosine diphosphate; AMP, adenosine monophosphate; ATP, adenosine triphosphate; BMI, body mass index; CCM, cirrhotic cardiomyopathy; ES, enrichment score; GLS; global longitudinal strain; LAVI, left atrial volume index; LV, left ventricular; MASLD; metabolic-dysfunction associated steatotic liver disease; Me2Py, N-methyl-2-pyridone-5-carboxamide; Me4Py, N-methyl-4-pyridone-5-carboxamide; MELD, Model for End-Stage Liver Disease; MeNAM, methylnicotinamide; NAD, nicotinamide adenine dinucleotide; NADP, nicotinamide adenine dinucleotide phosphate; NSBB, non-selective beta blocker; TR, tricuspid regurgitation.

We examined the plasma metabolome in ACLD patients using clinical and echocardiographic correlates. Plasma from adults with ACLD, collected in a prospective study (Sydney Local Health District Ethics Board; HREC/16/RPAH/701) was analyzed. Transthoracic echocardiograms (TTE) were performed within 6 months of blood collection [median 49 days; interquartile range (IQR) 11–104], using a Philips EPIQ system (X5-1 transducer, Philips Healthcare, Andover, MA, USA). CCM was defined by the 2020 CCM Consortium criteria (*Figure 1A*) for SD and advanced diastolic dysfunction (aDD). Patients meeting only two DD criteria were classified as indeterminate.

Metabolomic profiling was conducted using a hydrophilic interaction liquid chromatography (HILIC) and AMIDE analysis on an Agilent 1260 Infinity (Santa Clara, CA, USA) liquid tandem mass spectrometry (LC-MS/MS) system coupled to a QTRAP5500 mass spectrometer (ABSciex, Foster City, CA, USA). Metabolite concentrations were scaled to standardize variance. Fold changes (FC) < 1 indicate lower, and FC >1 higher, metabolite levels than the comparison group. Pathway analysis was performed using FGESA on nicotinate and nicotinamide metabolism annotated in the Small Molecules Pathways Database.^4^ Statistical analyses included chi-squared test for categorical variables, *t*-tests or Mann–Whitney *U* tests for continuous variables. Differential abundance analysis between the CCM and non-CCM groups, as well as metabolite-measurement association analyses, were conducted using moderated *t*-tests, adjusted for age, metabolic-dysfunction associated steatotic liver disease (MASLD), diabetes and hypertension. Statistical significance was set at *P* < 0.05. Research adhered to the Declarations of Helsinki and Istanbul. Written consent was obtained from all subjects.

Among 47 patients with ACLD median Model for End-Stage Liver Disease (MELD) score 16 (IQR 12–22); median Child-Pugh 10 (IQR 7–12), CCM was identified in 10 (21%), five with SD and five with aDD. Indeterminate diastolic function was found in 12 (26%). No significant baseline differences were observed between CCM and non-CCM patients (*Figure 1B*). Female sex was associated with DD [OR 5.0 (95% CI, 1.4–18.1); *P* = 0.01], but not CCM. Notably, CCM was unrelated to classical HFpEF risk factors (age, diabetes, hypertension, obesity, or MASLD, *Figure 1B*), suggesting the cardiac dysfunction observed was distinctly CCM, rather than classical HFpEF.

We uniquely describe the downregulation of NAD^+^ pathway intermediates and derivatives in patients with CCM, implicating the potential role of mitochondrial energy depletion in its pathogenesis. Pathway analysis (*Figure 1C*) of CCM vs. non-CCM patients found a negative normalized enrichment score (NES) for the nicotinic and nicotinamide (NAM) pathway (NES −1.65; *P* = 0.02), and downregulation of several key metabolites. The same pathway was inversely associated with septal mitral inflow early diastolic (E) velocity to septal early diastolic mitral annular (e′) velocity ratio (E/e′) (NES −1.90; *P* = 0.003). Individual metabolite analysis showed that septal e′ positively correlated with nicotinate (FC 1.29, *P* = 0.01) and nicotinic acid mononucleotide (NaMN) (FC 1.20; *P* = 0.03) (*Figure 1D*). Reduced septal e′ signifies impaired LV relaxation, so lower levels of these NAD^+^ metabolites were associated with DD. Left ventricular ejection fraction (LVEF) and left atrial volume index (LAVI) showed borderline associations with nicotinic acid, a conjugate base of nicotinate, with FCs of 1.48 (*P* = 0.06) and 0.79 (*P* = 0.06) respectively. This suggests a possible reduction associated with systolic *and* diastolic dysfunction.

Nicotinic acid and nicotinate are dietary substrates for the Preiss-Handler pathway, a major NAD+ biosynthetic route. NaMN is generated, then transformed into nicotinic acid adenine dinucleotide (NaAD), eventually forming NAD^+^. These results suggest a lack of NAD^+^ precursors in DD patients. Me2PY, along with Me4Py, are byproducts of methylnicotinamide (MeNAM) in the NAD salvage pathway and were downregulated in the pathway analysis. This implicates the downstream NAD pathway in DD as well as the aforementioned precursors.

These findings parallel emerging evidence of NAD+ depletion in HFpEF^5^ and, for the first time, link similar metabolic dysfunction to CCM. NAD^+^ deficiency-associated mitochondrial protein hyperacetylation has been proposed as a mechanism driving DD.^3,5,6^ This occurs through downregulation of sirtuin 3,^3,5^ a key mitochondrial deacetylase that plays a critical role in regulating mitochondrial protein acetylation levels, and regulating enzymatic activities that maintain basal cardiac function and stress responsiveness, for example, fatty acid oxidation.^5^ NAD^+^ supplementation has been explored as a therapeutic strategy.^5,6^ Our group recently demonstrated the benefits of NAD^+^ repletion with nicotinamide riboside (NR) in murine HFpEF and human myocardium in vitro,^3^ expanding on previous studies.^5^ Our results underscore the translational potential of NAD^+^ repletion in CCM.

We also found a novel association between elevated citraconate and DD, with levels increasing with E/e′ (FC 1.36; *P* = 0.049). Citraconate is a known inhibitor of itaconate, which suppresses succinate dehydrogenase, leading to succinate accumulation; succinate is a known non-septic metabolic driver of inflammation in cardiomyopathy and heart failure.^7^ Increasing citraconate levels have been proposed as a compensatory response to mitigate elevated itaconate levels; this finding suggests another potentially unexplored mechanism in CCM.

Phosphocreatine levels were inversely correlated with heart rate (HR) (FC 0.61, *P* = 0.03). Reduced HR variability is a hallmark of cirrhosis and an important prognostic indicator of decompensation risk.^8^ Our findings suggest a possible depletion of cardiac energy stores, offering new insight into the chronotropic incompetence associated with CCM, particularly in the setting of physiological stress.

Consistent with longstanding hypothesized mechanisms in CCM, we found elevated levels of the secondary bile acid taurodeoxycholate in patients with CCM (FC 1.96, *P* = 0.005). In animal models,^9^ bile acids impair cardiac contractility by switching isoforms of myosin heavy chains, reducing cardiac fatty acid oxidation and disrupting calcium homeostasis. Ours is among the the first human studies showing a bile acid metabolite association in CCM. We also observed a borderline association with increased levels of the endocannabinoid anandamide in CCM patients (FC 1.20, *P* = 0.058). Increased levels of endocannabinoids have been shown in rat models to induce negative inotropy^10^ and are a postulated driver of CCM; this is an exciting finding to replicate in humans.

In conclusion, this study describes the potential perturbation of the NAD^+^ pathway in CCM, providing evidence of an underlying metabolic disruption in this condition. These findings support the concept of a distinct ‘metabolomic fingerprint’ for CCM, which may lead to similar diagnostic and therapeutic advances as seen in HFpEF.

This study has several limitations. The sample size was small, and validation in larger cohorts is required. However, the observed associations align with existing literature. The median time between TTE and blood sampling was 49 days; simultaneous assessment would be ideal. Although plasma samples were optimally stored for metabolomic analysis, phosphocreatine degrades rapidly, warranting validation with snap-frozen samples. Finally, while plasma metabolomics provides valuable systemic insights, direct myocardial assessment is needed to confirm NAD+ depletion at the tissue level. Nonetheless, these findings establish a critical foundation for future targeted research.

## Authors’ contributions

M.G., J.F.O., A.M., I.W., and G.W.M. conceived of and designed the study. M.G., S.M., M.M., and I.H. collected data. R.P.L. and J.F.O. performed the laboratory analyses. M.G., G.G., and E.S. performed statistical analyses. M.G., J.F.O., G.W.M. and A.M. drafted the manuscript and approved the final version.

## Data Availability

Data available on request under consideration of privacy restrictions regarding living subjects.
